# Single-cell morphological responses as predictors of ciprofloxacin resistance in *Acinetobacter baumannii*

**DOI:** 10.1128/spectrum.00296-26

**Published:** 2026-08-17

**Authors:** Taechatam Malithong, Rubsadej Suwansaeng, Pornsawan Cholsaktrakool, Kornthara Kawang, Thanadon Samernate, Htut Htut Htoo, Filosofia F. T. A. Prasasti, Voraphoj Nilaratanakul, Vorrapon Chaikeeratisak, Poochit Nonejuie

**Affiliations:** 1 Institute of Molecular Biosciences, Mahidol University 98841, Nakhon Pathom, Thailand; 2 Program in Bioinformatics and Computational Biology, College of Interdisciplinary and Integrative Studies, Chulalongkorn University 26683, Bangkok, Thailand; 3 Excellence Center for Infectious Diseases, King Chulalongkorn Memorial Hospital, Thai Red Cross Society 105176, Bangkok, Thailand; 4 Division of Infectious Diseases, Department of Medicine, Faculty of Medicine, Chulalongkorn University and King Chulalongkorn Memorial Hospital, Thai Red Cross Society 26683, Bangkok, Thailand; 5 Department of Biochemistry, Faculty of Science, Chulalongkorn University 543911, Bangkok, Thailand; University of Saskatchewan, Saskatoon, Canada

**Keywords:** bacterial cytological profiling, susceptibility testing, antibiotic resistance

## Abstract

The rise of antimicrobial resistance (AMR) coincides with a declining pipeline of new antibiotics, underscoring the urgent need for rapid and accurate antibiotic susceptibility testing (AST). Single-cell AST approaches offer the potential to shorten diagnostic turnaround times; however, reliably distinguishing antibiotic-sensitive from antibiotic-resistant strains at the single-cell level remains challenging. In this study, we employed bacterial cytological profiling (BCP), a single-cell imaging technique previously used for mechanism of action identification, to quantitatively analyze the morphological responses of ciprofloxacin (CIP)-sensitive and CIP-resistant *Acinetobacter baumannii* strains, including both laboratory and clinical isolates. Upon CIP treatment, resistant strains generally exhibited a significantly reduced degree of morphological change compared with sensitive strains, independent of their resistance level. We then leveraged these differences in the degree of morphological response to develop a phenotypic discrimination framework for future AST applications. Using the reference strain AB5075 and six clinical isolates, this approach successfully distinguished sensitive from resistant phenotypes in this pilot set of strains with an accuracy of 0.93. These findings highlight the potential of single-cell morphology-based analysis as a promising foundation for the development of next-generation, rapid AST platforms.

**IMPORTANCE:**

Antimicrobial resistance represents a growing challenge for clinical management, in part because current antibiotic susceptibility testing can take days to determine which antibiotics will work. This study demonstrates that early, single-cell morphological responses to antibiotic exposure can serve as informative phenotypic indicators of resistance. By quantitatively analyzing how individual bacterial cells respond to antibiotic treatment, we show that resistant and susceptible strains can be reliably distinguished within 1 h of antibiotic exposure. Importantly, this approach remains effective across multidrug-resistant and clinical isolates. Together, these findings highlight rapid, imaging-based, single-cell phenotypic responses as a promising complement to current approaches for the future development of faster and more informative antimicrobial susceptibility diagnostics.

## INTRODUCTION

The rise of antimicrobial resistance (AMR) poses a serious global threat, affecting countries across all income levels and undermining the effectiveness of commonly used antibiotics for treating bacterial infections. Consequently, mortality rates associated with such infections are expected to increase (1, 2). The World Health Organization (WHO) has identified several bacterial species of particular concern that demand rigorous surveillance. Among these, the high prevalence of multidrug-resistant (MDR) strains of *Enterococcus* spp., *Staphylococcus aureus*, *Klebsiella* spp., *Acinetobacter baumannii*, *Pseudomonas aeruginosa*, and *Enterobacter* spp., collectively known as the “ESKAPE” pathogens, represents a critical challenge. Notably, MDR *A. baumannii* has been classified as a “critical priority” pathogen on the WHO list, as most clinical isolates exhibit resistance to multiple antibiotics, including those considered last-resort antibiotics (3).

Although the demand for new antibiotics continues to rise, the discovery of novel antimicrobial agents has steadily declined, underscoring the shortfall of the current antibiotic development pipeline to keep pace with escalating resistance (4, 5). Antimicrobial stewardship, therefore, remains one of the key strategies to mitigate the spread of AMR (6). Rapid and accurate antibiotic susceptibility testing (AST) is becoming more essential, as it enables timely and targeted therapeutic interventions. Conventional AST methods, including disk diffusion, E-test strips, and microdilution assays, are robust but limited by their time-consuming nature, often requiring multiple days to produce results. Although automated AST systems have improved throughput and reduced overall turnaround time, they still rely on population-level susceptibility profiles, overlooking the heterogeneous responses of individual cells and remaining relatively slow (7–9). Developing more accurate and rapid AST would ensure timely delivery of appropriate therapy, thereby reducing the risk of resistance development.

Image-based, single-cell analysis integrates high-resolution microscopy with computational pipelines to quantify numerous morphological features in individual cells across defined populations (10–12). In antibiotic discovery, image-based analysis has primarily been used to monitor morphological responses under exposure to drugs with known mechanisms of action (MoA) (13–16), allowing qualitative classification of MoA for new compounds based on morphological profiles. This approach has also been applied to investigate bacterial resistance, providing insights into antibiotic susceptibility profiles across multiple species, including *Staphylococcus aureus*, *Salmonella* Typhimurium, and *Escherichia coli* (17–20). While initial efforts have begun linking single-cell morphological profiling with AMR, the relationship remains poorly defined. Conventional readouts, such as MIC values and clinical breakpoints, provide well-established thresholds for population-based susceptibility interpretation (21). However, whether these conventional metrics can be directly translated to single-cell, image-based susceptibility testing remains unclear. This limitation arises in part from the inherent variability in bacterial responses to antibiotics at the single-cell level, which makes it technically challenging to clearly distinguish between susceptible and resistant phenotypes (14, 22–24). Thus, a better understanding of the morphological responses of resistant strains will provide crucial information for developing the accurate single-cell phenotypic AST.

Here, we used bacterial cytological profiling (BCP), a single-cell imaging approach for MoA identification, to examine the morphological responses of ciprofloxacin (CIP)-sensitive and -resistant strains at the reference MIC of a susceptible strain in order to evaluate whether this concentration could serve as a threshold for early binary discrimination between susceptible and resistant phenotypes at the single-cell level. In this study, we found that, upon CIP treatment, resistant strains generally exhibited less degree of morphological change than their sensitive counterparts, regardless of resistance level. Interestingly, and in contrast to previous reports, some resistant strains displayed pronounced morphological changes upon antibiotic exposure. Finally, by applying morphology-based analysis to the multidrug-resistant *A. baumannii* AB5075 model strain and six clinical isolates, we demonstrate that this approach can classify CIP susceptibility within the specific cohort examined.

## RESULTS

### Identifying morphological features relevant for distinguishing untreated and ciprofloxacin-treated cells

**Fig 1:**
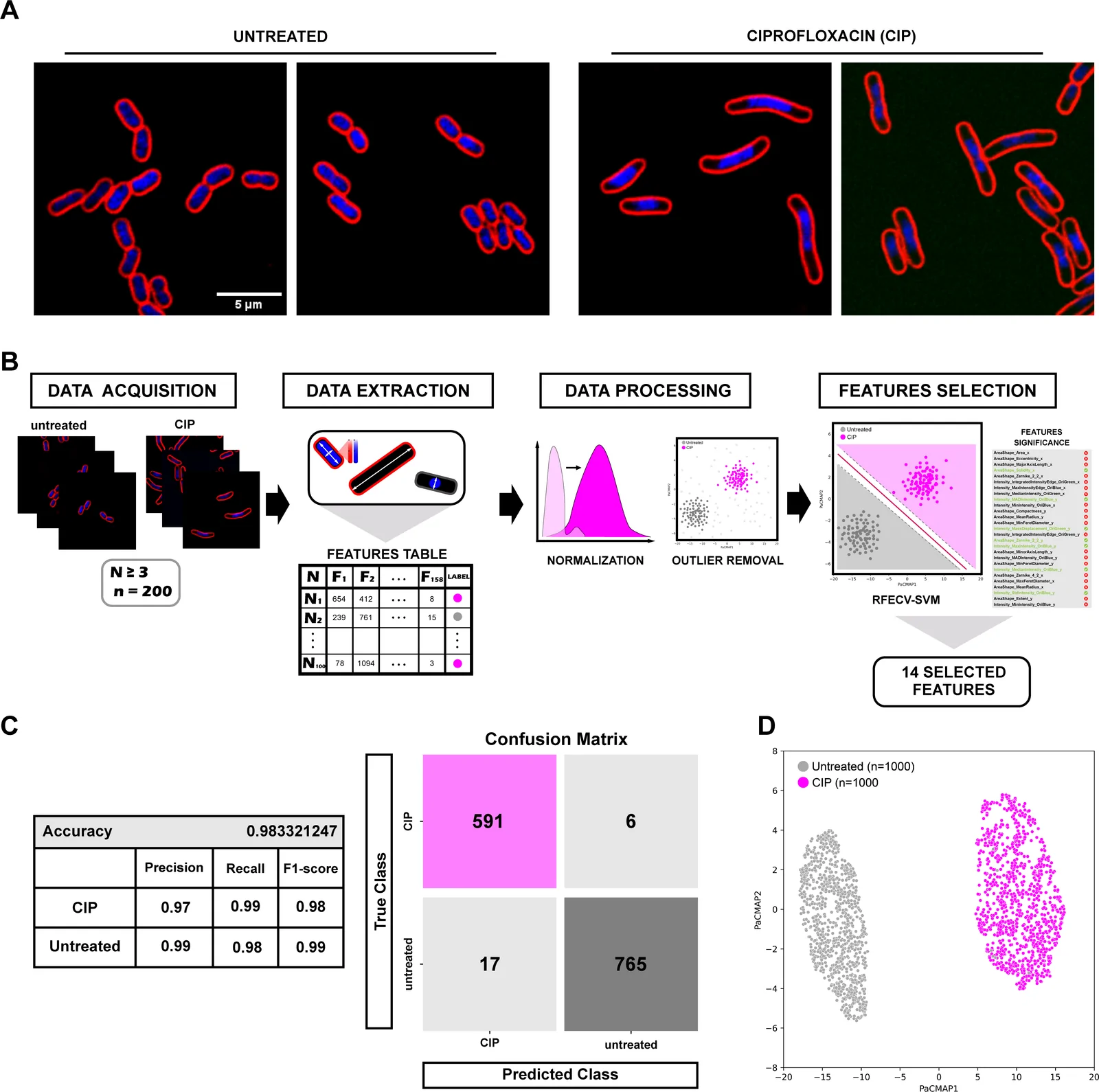
CIP-treated and untreated *A. baumannii* ATCC 17978 cells were distinguishable using 14 selected morphological features in this study. (**A**) Representative images of untreated and 0.5 μg/mL CIP-treated *A. baumannii* ATCC 17978 cells. In all images, cell membranes were stained with FM4-64 (red), nucleoids with DAPI (blue), and SYTOX Green (green). Scale bar: 5 μm. (**B**) Image-analysis pipeline for cell-feature extraction beginning with image acquisition, followed by data extraction, data processing, and feature selection using the RFECE-SVM machine-learning model. (**C**) Precision table and confusion matrix of single-cell profile classification using 14 features selected by RFECE-SVM. The colors in the confusion matrix represent the prediction outcomes: pink denotes true positives for CIP; dark gray denotes true negatives for untreated; and light gray denotes false predictions. (**D**) PaCMAP plot of *A. baumannii* ATCC 17978 cells treated with 0.5 μg/mL CIP compared with untreated cells visualized by compressing the selected 14 high-dimensional morphological features into a two-dimensional representation. In this plot, each point represents a single cell.

First, we sought to establish an analysis pipeline capable of distinguishing untreated from CIP-treated cells. A previous study examining the single-cell morphological responses of *A. baumannii* American Type Culture Collection (ATCC) 17978 to antibiotics at their respective MIC levels reported that 62 morphological features were sufficient for MOA classification across eight different antibiotics. The classification accuracy varied slightly depending on the degree of morphological change induced by each antibiotic. Importantly, that study also demonstrated that selecting relevant morphological parameters tailored to a specific data set improved classification accuracy (14). Building on this concept, we sought to identify the most relevant morphological features for distinguishing untreated from CIP-treated cells at the MIC level (0.5 µg/mL). First, fluorescence microscopy was performed on untreated cells and cells treated with 1× MIC of CIP. The results showed that CIP-treated cells exhibited morphological changes consistent with previously reported profiles (14) characterized by cell elongation and nucleoid localization at the mid-cell (Fig. 1A). Then, 3,000 individual cell profiles were randomly selected from at least three independent experiments for both untreated and CIP-treated conditions, as detailed in Materials and Methods. This data set was then subjected to feature selection in order to identify the most relevant features via RFECV-SVM approach as previously described (14) (Fig. 1B). From the total of 158 features extracted from image analysis, 14 features (Table S1) were selected for model construction to classify untreated versus CIP-treated cells, achieving an overall classification accuracy of 98.33% (Fig. 1C), which is slightly higher compared to the previous study (14). To further visualize the distribution and clusters of single-cell profiles, we applied PaCMAP dimensionality reduction using the selected features. As expected, CIP-treated cells were clearly separated from untreated cells (Fig. 1D). Collectively, these results demonstrate that a refined subset of 14 morphological features is sufficient to distinguish CIP-treated cells from untreated controls.

### CIP-resistant isolates exhibit morphological response variation upon CIP treatment

**Fig 2:**
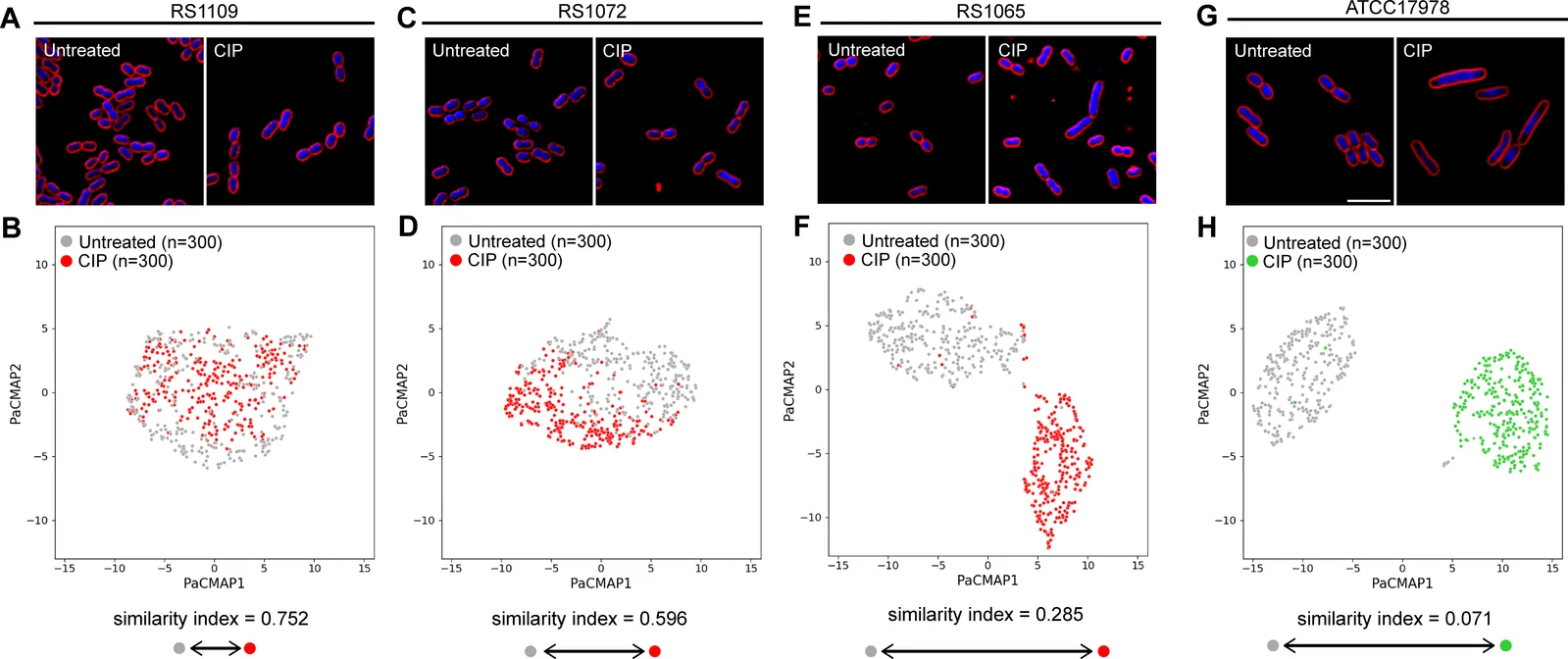
CIP-resistant isolates exhibit morphological response variation upon CIP treatment. Representative images of untreated and 0.5 μg/mL CIP-treated *A. baumannii* cells from strains RS1109 (**A**), RS1072 (**C**), RS1065 (**E**), and ATCC17978 (**G**). The untreated control image of ATCC17978 shown in Fig. 2G was reused from Fig. 1A because it represents the same representative untreated morphology. In all images, cell membranes were stained with FM4-64 (red), nucleoids with DAPI (blue), and with SYTOX Green (green). Scale bar: 5 μm. (**B, D, F, and H)** PaCMAP plots and similarity indices of untreated and 0.5 μg/mL CIP-treated *A. baumannii* cells from strains RS1109, RS1072, RS1065, and ATCC17978, respectively. Each data point represents an individual cell; a minimum of 300 individual cells (*n* ≥ 300) were evaluated per condition.

Next, we further asked if CIP-sensitive and -resistant *A. baumannii* strains exhibit any morphological response difference upon 0.5 μg/mL CIP treatment. However, it is well documented that genetic differences can display distinct morphological characteristics (25, 26). To minimize the influence of genetic background on morphological analysis in our proof-of-concept experiment, we isolated CIP-resistant strains derived from a single parental strain of *A. baumannii* ATCC 17978. In total, 25 CIP-resistant strains were obtained, exhibiting MIC values ranging from 4 to 32 μg/mL (Table S2). We next examined the extent to which these CIP-resistant strains respond morphologically to CIP at 0.5 μg/mL. Fluorescence microscopy, followed by image analysis using the 14 morphological parameters, revealed that the majority of resistant strains exhibited little to no morphological changes, resulting in overlapping profiles with untreated cells (RS1109, Fig. 2A and B). This finding corresponds with the previous study where resistant strains exhibited no morphological changes upon the antibiotic treatment (20). However, some resistant strains displayed a certain degree of morphological alterations upon treatment, resulting in non-overlapping clusters with the untreated group (RS1072, Fig. 2C and D). Interestingly, a few resistant strains showed drastic morphological changes, forming a clear separation between treated and untreated profiles (RS1065, Fig. 2E and F), similar to those observed in *A. baumannii* ATCC 17978 (Fig. 2G and H). These observations prompted us to quantify the degree of morphological change between untreated and CIP-treated cells using a similarity index, as previously described (14). A lower similarity index indicates a clearer separation of morphological clusters and, therefore, reflects a stronger effect of antibiotic treatment on bacterial morphology. As expected, the CIP-sensitive *A. baumannii* ATCC 17978 strain, which exhibited a clear separation between untreated and CIP-treated cells, resulted in a low similarity index of 0.071 (Fig. 2H). In contrast, the CIP-resistant RS1109 strain showed a substantial overlap between treated and untreated profiles, resulting in a high similarity index of 0.752 (Fig. 2B). RS1072 and RS1065 followed the same trend, showing progressively lower similarity indices of 0.596 and 0.285, respectively, consistent with increased separation between the two morphological clusters (Fig. 2D and F). This finding contrasts with the previous report, which assumed that resistant isolates generally exhibit no morphological response to antibiotic treatment (20).

### CIP-resistant strains exhibit less degree of morphological change upon CIP treatment when compared with their sensitive counterparts

**Fig 3:**
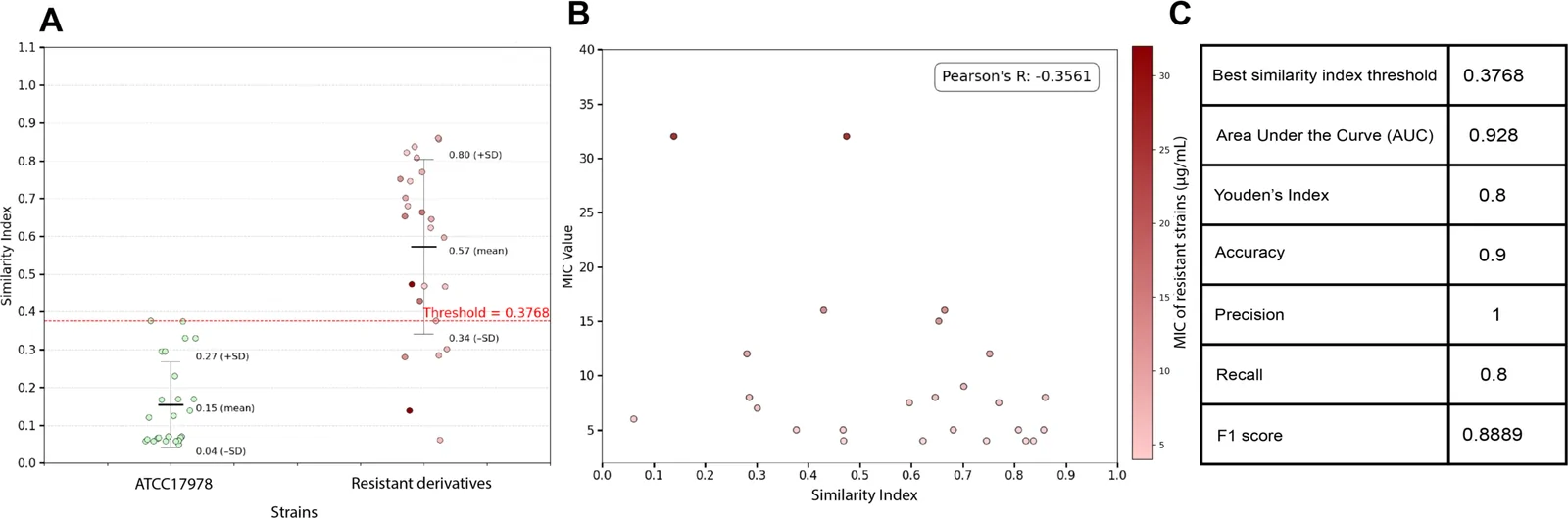
CIP-resistant strains exhibit less degree of morphological change upon CIP treatment, regardless of their CIP MIC level, when compared with their sensitive counterparts. (**A**) Summary of the similarity index between untreated and CIP-treated of all 25 CIP-resistant strains in comparison with *A. baumannii* ATCC 17978. Green dots indicate strains classified as CIP-sensitive, and red dots indicate CIP-resistant strains according to CLSI criteria. The dark horizontal line indicates the population mean, and whiskers represent the standard deviation (SD). Each data point represents a single similarity index value derived from comparing a random sub-sample of 200 treated and 200 untreated cells. For the ATCC 17978 reference strain, data points represent *n* = 25 independent biological replicates. For the resistant group, data points represent *n* = 25 distinct clinical isolates. (**B**) Scatter plot of MIC values and similarity index analysis of 25 CIP-resistant strains. (**C**) Precision table of sensitive and resistant classification using similarity index threshold at 0.3768, yielding an AUC of 0.928 and 95% CI [0.840, 0.989].

The variation of morphological responses to CIP treatment observed in some CIP-resistant isolates (Fig. 2C and E) urged us to perform a comprehensive analysis of single-cell morphological responses by quantifying the degree of morphological change using a similarity index of all 25 CIP-resistant strains in comparison with *A. baumannii* ATCC 17978 upon CIP treatment. As expected, the average similarity index between untreated and CIP-treated *A. baumannii* ATCC 17978 cells clustered around 0.15 ± 0.11, reflecting a drastic morphological shift caused by antibiotic perturbation in the sensitive strain. In contrast, resistant strains displayed significantly higher similarity indices (0.57 ± 0.23) (Fig. 3A), consistent with the observed minimal morphological alterations. Notably, the similarity indices among resistant strains were more variable than those of the sensitive strain; some resistant isolates exhibited similarity indices as low as those of sensitive cells, suggesting that while most resistant strains undergo limited morphological changes, their responses are not uniform.

### No correlation between degree of CIP resistance and extent of morphological changes

The previous study reported that the accuracy of single-cell phenotypic classification was influenced by both the relative CIP concentration used in the experiment and the inherent MIC of individual strains (20). In particular, the study reported that a higher proportion of CIP-resistant *E. coli* strains exhibited morphological changes when treated with CIP at concentrations near its MIC and hence identified as susceptible cells. Considering this, and given the variability in similarity indices observed among resistant strains in our study, we speculate if the higher MIC of the resistant strains correlated with the lower degree of morphological change or not. As expected, our analysis revealed no correlation between MIC values and similarity indices (Fig. 3B), indicating that the level of resistance does not determine the degree of morphological change upon treatment with 0.5 μg/mL CIP (Fig. 3B). Collectively, these results suggest that, under CIP treatment at 0.5 μg/mL, most CIP-resistant strains, regardless of their MIC level, exhibit minimal or no morphological changes compared to sensitive strains.

### Classification of sensitive and resistant strains via degree of morphological change

We next tested whether the degree of morphological change, as indicated by similarity index, could be applied to classify CIP-sensitive and -resistant strains using receiver operating characteristic (ROC) analysis to find the best similarity index threshold for binary classification (sensitive vs resistant). The analysis identified a classification threshold of similarity index between *A. baumannii* ATCC 17978 and its CIP-resistant derivatives at 0.3768, yielding an AUC of 0.928, 95% CI [0.840, 0.989], and an overall classification accuracy of 0.9 (Fig. 3C; Fig. S1). This suggests that the degree of morphological change could be leveraged to classify CIP-sensitive and -resistant strains. We also compared the performances of the 14 morphological features specifically selected in this study with the previously reported universal set of 62 features, which was originally optimized for classifying eight different antibiotic classes. Re-analysis of all extracted data using the 62-feature set produced an AUC of 0.8672, 95% CI [0.7516, 0.9573], and an overall classification accuracy of 0.84 (Fig. S2), indicating lower efficiency compared with the 14-feature model. Thus, the CIP-specific features outperformed the universal set, as reflected by improved accuracy when using the 14 selected parameters from this study.

### Degree of morphological change potentially enables the classification of CIP susceptibility profile in the MDR-*A. baumannii* and clinical strains

**Fig 4:**
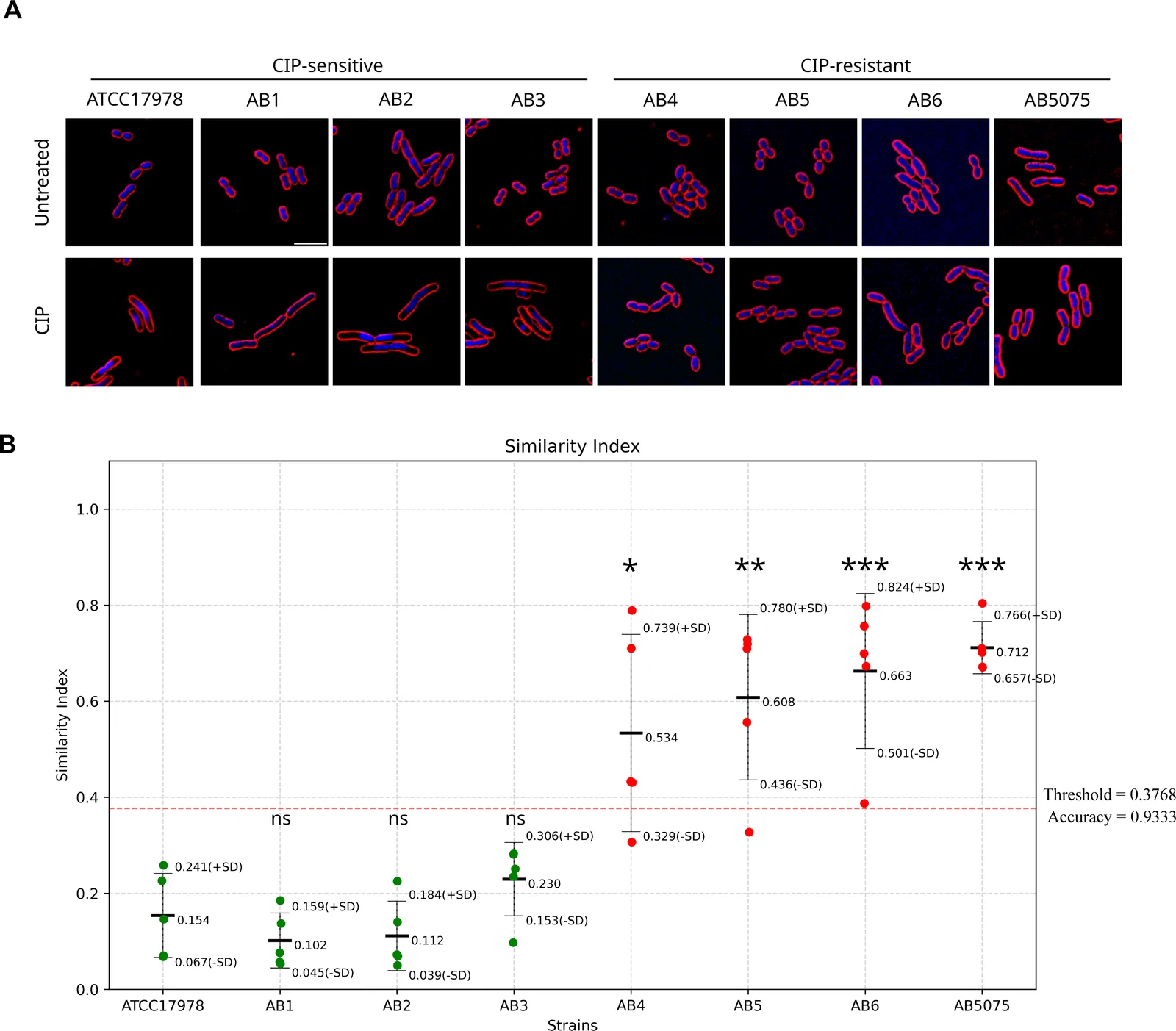
Degree of morphological change reveals CIP susceptibility profile of the MDR-*A. baumannii* strains. (**A**) Representative images of untreated and 0.5 μg/mL CIP–treated *A. baumannii* cells from clinical strains AB1-6 and AB5075. In all images, cell membranes were stained with FM4-64 (red), nucleoids with DAPI (blue), and with SYTOX Green (green). Scale bar: 5 μm. (**B**) Summary of similarity index between untreated and CIP-treated of the ATCC 17978 laboratory reference strain, all clinical isolates AB1-6, and the AB5075 strain. Individual data points represent independent biological replicates (*n* = 5 for each strain). Green dots indicate strains classified as CIP-sensitive, and red dots indicate CIP-resistant strains according to CLSI criteria. The dark horizontal line represents individual strain means, and whiskers represent the standard deviation (SD). The red dashed horizontal line represents the similarity index threshold at 0.3768, achieving a classification accuracy of 0.9333. Statistical significance was determined using a two-tailed Welch’s unpaired *t*-test (assuming unequal variance) comparing each clinical isolate and the AB5075 strain against the ATCC 17978 reference strain: ns, non-significant; *, *P* < 0.05; **, *P* < 0.01; and ***, *P* < 0.001.

Having examined a morphology-based classification approach in *A. baumannii* ATCC 17978 and its CIP-resistant derivatives, we next applied the method to the multidrug-resistant strain AB5075 and six clinical isolates of *A. baumannii*. The antibiotic susceptibility profiles of these strains were known prior to analysis and used as ground-truth labels for evaluating model performance (Table 1). The rationale was to determine whether this approach could be extended beyond laboratory reference strains. First, whole-genome sequencing was performed on all six clinical strains to confirm that the isolates used in this study represented diverse genetic backgrounds relevant to antibiotic susceptibility. Genome analysis revealed that the clinical isolates harbored distinct predicted antibiotic resistance phenotypes (Table S3). Notably, AB1–3 carried only *β*-lactam-associated resistance determinants (e.g., *bla*_OXA-64_), likely reflecting the intrinsic resistance of *A. baumannii*, whereas AB4–6 harbored multiple predicted resistance phenotypes, including point mutations that confer CIP resistance. Then, morphological analysis revealed that AB1, AB2, and AB3 (CIP-sensitive) displayed pronounced morphological alterations upon treatment (Fig. 4A) and correspondingly low similarity indices (Fig. 4B). In contrast, clinical isolates AB4, AB5, and AB6, and AB5075 (CIP-resistant) exhibited less pronounced morphological changes (Fig. 4A) and hence high similarity indices (Fig. 4B). Notably, although AB4, AB5, and AB6 all showed the same MIC values against CIP (128 μg/ml), the average similarity index varied among them. This observation further supports our earlier finding in laboratory strains that there is no direct correlation between MIC level and the degree of morphological change. Finally, applying the classification threshold of 0.3768 successfully distinguished sensitive from resistant phenotypes across all six clinical isolates with an overall accuracy of 0.93 (Fig. 4B). Collectively, these results demonstrate that morphological change-based analysis can provide reliable information on CIP resistance phenotypes in clinical isolates of *A. baumannii*.

**TABLE 1:** Clinical isolates of *A. baumannii* used in the study and their antibiotic susceptibility profiles

Strains	Type of specimens	NCBI genome accession	Minimum inhibitory concentration (μg/mL)			
			Ciprofloxacin (CIP)	Minocycline (MIN)	Meropenem (MER)	Piperacillin (PIP)
AB5075 (27)	−^*c*^	Published elsewhere^*b*^	128 (R)	0.15625 (S)	16 (R)	128 (R)
AB1 (S-AB1)	Urine	CP196885 and CP196886	0.5 (S)	0.3125 (S)	0.3125 (S)	0.3125 (S^*a*^)
AB2 (S-AB2)	Hemoculture	CP196887–CP196890	0.5 (S)	0.3125 (S)	0.25 (S)	17 (S^*a*^)
AB3 (S-AB8)	Endotracheal aspirate	CP196891 and CP196892	0.5 (S)	0.3125 (S)	0.25 (S)	30 (S^*a*^)
AB4 (SP2391)	Sputum	JBPZNT000000000	128 (R)	1 (S)	4 (I)	68 (R^*a*^)
AB5 (A2006)	Sputum	CP196878–CP196884	128 (R)	0.625 (S)	4 (I)	68 (R^*a*^)
AB6 (A2005)	Sputum	CP196873–CP196877	128 (R)	1 (S)	4 (I)	68 (R^*a*^)

^*a*^ R/S was obtained from the antibiogram report since no CLSI breakpoint was available for piperacillin (PIP) against *A. baumannii*.

^*b*^ Previously published elsewhere.

^*c*^ "–" indicates that the specimen type is not applicable for AB5075.

Notably, we also observed that clinical isolates in the untreated condition exhibited basal morphological variation, defined as heterogeneity in cell shape, size, and other observable features prior to treatment (Fig. S3), suggesting that, under untreated conditions, different strains display different inherent morphologies. We then asked whether these baseline morphological characteristics could provide insights into the susceptibility profiles of the bacteria. Using the universal set of 62 morphological features to analyze untreated samples from six clinical isolates, we found that the untreated populations of AB1–3 (CIP-sensitive) clustered together, whereas AB4–6 (CIP-resistant) formed a distinct cluster separated from the sensitive group (Fig. S4*).* This observation is consistent with a previous study in *Salmonella* Typhimurium, which demonstrated that a predictive model could accurately classify ciprofloxacin susceptibility, even without antibiotic exposure (19).

## DISCUSSION

The morphological response of bacteria has long served as a key indicator for understanding their physiological states, dating back to the invention of the microscope. More recently, the advent of single-cell imaging and big data analysis has enabled bacterial cytological profiling at unprecedented resolution, thereby accelerating both antibiotic discovery and the development of AST methodologies (17, 19, 20, 28). Previous studies have shown that resistant strains generally do not exhibit morphological responses to antibiotics they are resistant to, suggesting that such characteristics could serve as indicators of bacterial susceptibility profiles (19, 20). However, establishing boundaries between susceptible and resistant strains using single-cell-based approaches remains at an early stage, and standardized criteria analogous to MIC or clinical breakpoints for image-based susceptibility testing have not yet been defined. In this study, we show that a CIP concentration corresponding to the MIC of a susceptible strain can serve as a threshold for distinguishing susceptible and resistant phenotypes at the single-cell level within the specific strain cohort examined. However, several important limitations affecting the biological interpretation and broader generalizability of this approach should be carefully considered and are discussed below.

The antibiotic concentration used to define the threshold for susceptible and resistant phenotype discrimination at the single-cell level represents a critical experimental parameter. A previous study showed that, within a resistant population, a higher proportion of susceptible cells exhibit morphological changes (indicative of susceptibility) when treated with antibiotic at concentrations near its MIC (20). This observation highlights the importance of selecting appropriate antibiotic concentrations when developing image-based AST methods. To avoid this ambiguity, certain image-based AST studies apply high antibiotic concentrations to ensure robust and consistent morphological readouts (17, 20). While this strategy is effective for training classification models, reliance on high concentrations risks false negatives when evaluating resistant isolates. In our study, CIP was tested at the reference MIC for *A. baumannii* ATCC 17978 (0.5 µg/mL). Although this concentration is lower than those used in prior studies (17, 19, 20), it may still be above the minimal level required to induce morphological changes. Indeed, a previous study has reported detectable morphological alterations at concentrations as low as 0.25 × MIC (29, 30). Thus, it is possible that treatment at 1 × MIC may still carry a higher likelihood of false negatives. Future work should therefore focus on identifying the minimum antibiotic concentration that induces measurable morphological changes, which may serve as a MIC-equivalent reference for single-cell morphology–based analyzes.

Another limitation is the lack of a clear linkage between genetic factors and phenotypic characteristics, particularly in the context of antibiotic-induced morphological changes. Although this proof-of-concept study used CIP-resistant strains derived from a single parental strain (*A. baumannii* ATCC 17978) to minimize the influence of genetic background, the underlying resistance mechanisms of all 25 induced CIP-resistant strains were not characterized at the genetic level. In the case of clinical isolates, although whole-genome sequencing was performed on all six isolates, the analysis was limited to *in silico* prediction of resistance-associated genes and did not directly investigate the influence of individual genomic variations on cellular morphology. As a result, it remains unclear whether the observed morphological responses of CIP-resistant strains to CIP treatment are driven by specific genetic determinants or by particular underlying resistance mechanisms. This may partly explain why, in this study, although CIP-resistant strains generally exhibited a lower degree of morphological change upon CIP treatment regardless of resistance level, a subset of resistant strains still displayed pronounced morphological changes. This observation suggests that underlying resistance mechanisms may contribute to variability in morphological responses within the resistant population.

In particular, a previous study using an imaging-based approach to infer CIP susceptibility in *Salmonella* Typhimurium demonstrated that different mutations associated with CIP resistance could influence bacterial morphological responses both in the presence and absence of CIP exposure (19). Moreover, other studies have also demonstrated that cellular proximity can drive single-cell variation in antibiotic resistance through local differences in antibiotic concentrations mediated by efflux pump activity (31, 32). Such microenvironmental effects may lead to uneven intracellular antibiotic concentrations among neighboring cells, whereby a subset of cells within a resistant population may still experience sufficiently high antibiotic levels to induce morphological changes. Consequently, whether the genetic basis of resistance mechanisms influences morphological responses under antibiotic stress requires further investigation.

Regarding the use of basal morphology (untreated cells) for inferring susceptibility, we observed that CIP-resistant clinical isolates exhibited distinct basal morphologies compared with sensitive isolates (Fig. S4), suggesting that inherent morphological traits may serve as indicators of antibiotic susceptibility. This finding aligns with previous studies in *Salmonella* Typhimurium (19) and *E. coli* (28), which demonstrated that predictive models could accurately classify antibiotic susceptibility even in the absence of antibiotic exposure, based solely on basal bacterial morphology. However, both our findings in *A. baumannii* and these previous studies remain limited by the small data set sizes, which may increase the risk of model overfitting. Future work should therefore aim to expand morphology-based analyzes across a broader range of clinical isolates, resistance mutations, and bacterial species to establish a more generalizable framework for single-cell morphology-based AST development.

Another limitation worth noting here is that our study primarily focused on CIP as a proof of concept. This choice was based on three reasons: 1) its ability to induce drastic and relatively homogeneous morphological changes upon treatment, 2) the ease of generating resistant isolates in the laboratory, and 3) the high prevalence of CIP resistance among clinical strains. Nevertheless, other antibiotic-specific morphological responses should be further explored. It is possible that the current method may require optimization for other antibiotics, particularly those that induce multiple or heterogeneous morphological responses (i.e., aminoglycoside), or those that target the bacterial membrane (i.e., colistin), where a specific concentration threshold is often required to trigger detectable morphological changes. Another limitation is that this study used *A. baumannii* as the sole model organism. Whether the same approach can be extended to other bacterial species remains to be investigated. However, previous studies in various Gram-negative bacteria (13, 33–35), Gram-positive bacteria (36, 37), and even *Mycobacterium tuberculosis* (38) have reported similar antibiotic-induced morphological responses, suggesting that this morphology-based strategy could potentially be applied to AST development for other pathogens.

Regarding future AST development, numerous studies have employed single-cell imaging technologies to monitor antibiotic susceptibility patterns at the individual-cell level, enabling more rapid susceptibility readouts (17, 19, 20, 28, 39, 40). Our study demonstrates that this approach does not require prior knowledge of resistance profiles from the tested strains. Instead, it quantifies the degree of morphological change within the same strain before and after antibiotic exposure. Although basal morphological variation was observed among individual cells in some clinical isolates, it did not interfere with the interpretation of CIP-induced changes and, therefore, did not affect susceptibility classification. However, it is important to note that although the similarity index threshold of 0.3768 derived from this study could be applied to identify the susceptibility profiles of AB5075 and the six clinical isolates, this threshold should be considered specific to the experimental cohort and conditions evaluated here. Given the limited sample size and the potential influence of experimental variables, it should not yet be interpreted as a universally transferable threshold applicable across different strain collections, laboratories, or imaging platforms. Therefore, broader generalization remains premature. Future studies should expand the validation across more diverse strain collections. In particular, inclusion of additional laboratory reference strains (e.g., ATCC 19606, AYE, and ACICU) as well as well-characterized clinical isolate collections with defined genetic backgrounds would be necessary to improve the robustness and generalizability of the similarity index-based method. Nonetheless, we acknowledge that differences in instrumentation and experimental setup may also introduce systematic variation, and future studies will be required to evaluate the robustness of this approach across different experimental platforms.

Furthermore, incorporating single-cell tracking to continuously monitor morphological dynamics could allow real-time determination of bacterial susceptibility. This capability would be particularly valuable in polymicrobial infections, where individual cells or even different species within the same sample, may exhibit distinct susceptibility profiles or influence each other’s antibiotic responses (41–43). Overall, such a single-cell morphology-based approach holds great promise for advancing next-generation AST development.

## MATERIALS AND METHODS

### Bacterial strains

*A. baumannii* ATCC17978, AB5075, and six clinical isolates were used in this study. The six clinical isolates were derived from the Microbiology Laboratory of King Chulalongkorn Memorial Hospital under research project COA No. 1559/2021 approved by the Institutional Review Board (IRB) of the Faculty of Medicine, Chulalongkorn University, Bangkok, Thailand (IRB No. 613/62).

### DNA extraction and genome assembly

#### Specimen collection and subculture

*A. baumannii* clinical isolates were obtained from various specimen types, including sputum, blood, urine, and endotracheal aspirates (Table 1). The isolates were subcultured on LB agar plates and in LB broth at 37°C overnight with shaking at 200 rpm prior to whole-genome sequencing.

#### Genomic DNA extraction

Genomic DNA was extracted using the Quick-DNA HMW MagBead Kit (Zymo Research, Cat no. D6060). Briefly, cells were harvested by centrifugation at 5,000 × *g* for 1 min, and the resulting pellets were lysed with SDS and Proteinase K. DNA was recovered by magnetic-bead-based purification and subjected to secondary cleanup with AMPure XP beads. DNA quality and purity were assessed using Nanodrop spectrophotometry (target A260/A280 ≈ 1.8), and DNA concentration was accurately quantified with the Qubit dsDNA high-sensitivity assay.

#### Nanopore library preparation and sequencing

Nanopore (44) library preparation was performed using the Rapid Barcoding Kit (SQK-RBK004; Oxford Nanopore Technologies). Each sample received a unique barcode, followed by pooling, AMPure XP bead cleanup, and adapter attachment. The MinION flow cell (FLO-MIN106 R9.4.1) was primed according to ONT protocols, and the final library mix was loaded for sequencing in the Oxford Nanopore MinION Mk1B sequencer for 72 h.

#### Bioinformatics: quality control, assembly, and annotation

We basecalled all FAST5 files using Guppy v6.1.7 in super-high accuracy mode (minimum Phred score Q ≥ 10) (45), which included demultiplexing and barcode/adaptor trimming. Raw read quality was assessed with pycoQC (46) v2.5.3.0, FastQC v0.11.9 (47), and NanoPlot v1.40.0 (48), capturing metrics, such as total bases, read counts, read-length distribution, N50, and Phred quality scores. Low-quality regions were filtered out by trimming 50 bp from both ends of each read with NanoFilt v2.8.0 (48) while retaining only reads at least 200 bp long (49). Genome assembly and polishing were performed with Flye v2.9.3-b1797 (50) and Medaka v1.8.0 (https://github.com/nanoporetech/medaka), respectively. Taxonomic classification was conducted using Kraken2 (51) to confirm species identity and detect possible contamination. Assembly completeness, contamination, and structural continuity were evaluated using BUSCO v5.4.3 (52, 53) and CheckM2 v1.0.2 (54). Genome annotation was carried out with Bakta v1.11.0 (55), and antimicrobial resistance (AMR) determinants were identified using StarAMR v0.5.1 (56) against the ResFinder database (downloaded on 26 January 2024). The final assemblies were uploaded to National Center for Biotechnology Information (NCBI) (Table 1).

A detailed description of the command-line, relevant parameter settings, and software/source links used for the genome assembly and downstream analyses is provided in Table S4.

### Minimum inhibitory concentrations (MICs)

MICs were determined using the broth microdilution method as previously described (14), with results shown in Table 1. Briefly, cells were cultured in LB broth to an optical density of 0.2 at 600 nm (OD600). The cell cultures were subsequently diluted 1:100 into 96-well plates containing differing concentrations of each antibiotic in LB medium. MIC values were determined after 24 h of incubation at 30°C and interpreted according to the Clinical and Laboratory Standards Institute (CLSI) breakpoint criteria (21).

### CIP-resistance isolation

Twenty-five non-clinical CIP-resistant isolates were generated from *A. baumannii* ATCC 17978 using two distinct induction methods (18, 57). The first method involved selection on agar plates. Briefly, individual *A. baumannii* colonies were cultured overnight in 5 mL of LB broth at 30°C. Aliquots (250 μL) of the overnight culture were then spread onto LB agar plates containing ciprofloxacin at concentrations 2 to 5× the MIC. Plates were incubated at 30°C for up to 7 days until growth was observed. The second method utilized serial passaging in 96-well plates with incrementally increasing ciprofloxacin concentrations (0.2× to 8× MIC). Briefly, overnight cultures were diluted 1:100 and added to 96-well plates containing 0.2× MIC of ciprofloxacin in LB medium, followed by incubation at 30°C until bacterial growth was evident. Subsequently, the cultures were passaged into medium with a higher antibiotic concentration. This process was repeated until the bacteria demonstrated the ability to grow in medium containing 4 to 8× the MIC of ciprofloxacin. For strain confirmation, Leeds agar, a selective and differential medium for *Acinetobacter*, was used for primary identification, and then later confirmed 16S rRNA sequencing via colony PCR using the forward primer BSF8/20 (5′-AGAGTTTGATCCTGGCTCAG-3′) and the reverse primer REVB (5′-GGTTACCTTGTTACGACTT-3′).

### Fluorescence microscopy

*A. baumannii* cultures in the early log phase (OD600 of 0.2) were treated with 1× MIC (0.5 μg/mL) of CIP at 30°C in a roller. After 1 h of incubation, samples were collected prior to staining with a combination of fluorescent dyes consisting of 1 μg/mL FM 4-64, 2 μg/mL DAPI, and 0.5 μM SYTOX Green. The cells were harvested by centrifugation at 10,000 rpm for 1 min, and the cell pellet was resuspended in one-fifth of the original volume. Five microliters of the stained sample was transferred onto an agarose pad (1.2% agarose in 10% LB broth) and subsequently observed under a fluorescence microscope (DeltaVision Ultra microscope). Constant settings and imaging parameters were used throughout all experiments. The same representative untreated control image of *A. baumannii* ATCC 17978 was reused across multiple figures when illustrating identical experimental conditions.

### Image analysis

Image analysis was performed as previously described (14). Briefly, deconvolved images were first processed using FIJI (58), and then CellProfiler software (59) was employed to extract morphological features from individual cells prior to machine learning analysis. A total of 158 features were extracted for further analysis, aiming to characterize the unique features distinguishing CIP-treated from untreated *A. baumannii* reference strains. Briefly, cell profiles from three independent experiments for both treated and untreated conditions were randomly pooled, resulting in approximately 3,000 cell profiles for each condition. These profiles were then analyzed using the scikit-learn library. Single-cell profiles were normalized using a Quantile Transformer (60) and outliers, such as mis-segmented objects or unwanted cell debris (constituting 10% of the data), were removed using hierarchical density-based spatial clustering of applications with noise (HDBSCAN) (61). Morphological feature optimization was conducted using a recursive feature elimination with cross-validation based on a support vector machine (RFECV-SVM) model (62) to determine the optimal number of features. The model utilizing these selected features was then evaluated for its ability to distinguish morphological profiles between CIP-treated and untreated samples. The data set was partitioned into an 80:20 ratio for the training and testing sets, respectively. Evaluation metrics were used to indicate the performance of the model (63) statistically calculated from the confusion matrix as follows:

Accuracy is the metric representing the proportion of correctly predicted data instances over the total number of predictions.


$$Accuracy\;=\;\frac{TP\;+\;TN}{\left(TP\;+\;TN\;+\;FP\;+\;FN\right)}$$

Precision is the metric measuring the proportion of correctly predicted true positives out of all positive predictions.


$$Precision\;=\;\frac{TP}{\left(TP\;+\;FP\right)}$$

Recall is the metric measuring the proportion of correctly predicted true positives out of all actual true positives.


$$Recall\;=\;\frac{TP}{\left(TP\;+\;FN\right)}$$

The *f*1-score is the harmonic mean of precision and recall, providing a single metric that balances the trade-off between them.


$$f1-score\;=\;2\;\times \frac{Precision\;\times \;Recall}{Precision\;+\;Recall}$$

These metrics are based on true positive (TP), true negative (TN), false positive (FP), and false negative (FN) values.

Finally, Pairwise Controlled Manifold Approximation (PaCMAP) was applied to visualize the clustered data in a two-dimensional (2D) Euclidean space (64). For both non-clinical and six clinical isolates, at least 300 cells were randomly selected from the same batch. The selected morphological features were then used to generate PaCMAP plots on these data and determine a similarity index in a subsequent step, allowing for visualization and quantification of the differences between the treated and untreated profiles.

### Similarity index calculation

The similarity index is a metric that quantifies the similarity between two clusters; a higher index value indicates a greater degree of relatedness. In this study, the similarity index was used to quantify the morphological similarity between untreated and ciprofloxacin-treated cells. The index was calculated based on the Euclidean distances (visualized on the PaCMAP) between the untreated cluster centroid and 200 randomly selected CIP-treated cells. Cluster centroids were determined using the mean shift clustering algorithm, which identifies high-density regions within the scattered cell profile data as cluster centers, with each cluster required to contain more than 50 cells.


$$Similarity\;index\;=\;\frac{\sum_{i=1}^{\text{200}}\frac{1}{d\left(i,j\right)}}{200}$$

The variable *d*_*ij*_ represents the minimum Euclidean distance between an untreated cell and a ciprofloxacin-treated cell.

### Receiver operating characteristic (ROC)

ROC analysis was employed to determine the similarity index cut-off for differentiating between resistant and susceptible profiles (65, 66). This analysis calculated sensitivity and specificity to establish an optimal threshold. The ROC curve was generated by plotting the true positive rate (sensitivity) against the false positive rate (1 - specificity) across all possible threshold values. The optimal threshold represents the point with a balanced trade-off between these two metrics identified by varying the threshold to minimize the false positive rate (maximize specificity) while maintaining the true positive rate (sensitivity). This point is often calculated using the Youden index:

J = Sensitivity + Specificity − 1

The similarity indices from all sensitive (ATCC17978) and resistance isolates strains were subjected to ROC analysis to find the optimal threshold for differentiating the morphological responses of resistant and sensitive strains in the context of AST. The area under the ROC curve (AUC) obtained from this analysis (Fig. S1) reflects the overall discriminatory power of the test. A maximum AUC of 1.0 signifies perfect discrimination between the CIP-treated and untreated similarity indices with no overlap. Conversely, an AUC of 0.5 indicates no discriminatory ability beyond random chance. The 95% confidence intervals (95% CI) for the AUC analyses were estimated using bootstrap resampling with 2,000 iterations.

## Data Availability

The whole-genome sequencing data generated in this study have been deposited in the NCBI database under the accession numbers listed in Table 1. Detailed information for the genome assembly and downstream bioinformatics analyses, including the software tools, software versions, parameter settings, command-line instructions, and software/source links, is provided in Table S4. All source codes used for the morphological analysis pipeline, together with representative example data sets supporting the findings of this study, are publicly available on GitHub at https://github.com/Taechatam/BCP_Similarity_Pipeline, where brief instructions for the analysis are also provided.

## SUPPLEMENTAL MATERIAL

Supplemental figures and tables spectrum.00296-26-s0001.pdf Figures S1 to S4 and Tables S1 to S4.

ASM does not own the copyrights to Supplemental Material that may be linked to, or accessed through, an article. The authors have granted ASM a non-exclusive, world-wide license to publish the Supplemental Material files. Please contact the corresponding author directly for reuse.
