# Supplementary material for: Single-cell morphological responses as predictors of ciprofloxacin resistance in Acinetobacter baumannii
Source: Microbiol Spectr. 2026 Aug 17;14(10):e00296-26. doi: 10.1128/spectrum.00296-26 (PMC13689321; doi:10.1128/spectrum.00296-26)
Supplement: Supplementary file 1 [file spectrum.00296-26-s0001.pdf]

Supplemental material for

Title:
Single-Cell Morphological Responses as Predictors of Ciprofloxacin Resistance in *Acinetobacter baumannii*

Authors
Taechatam Malithong,[1] Rubsadej Suwansaeng,[1] Pornsawan Cholsaktrakool,[2,3] Kornthara Kawang,[3,4] Thanadon Samernate,[1] Htut Htut Htoo,[1] Filosofia F. T. A. Prasasti,[1] Voraphoj Nilaratanakul,[3,4] Vorrapon Chaikeeratisak,[5] Poochit Nonejuie[1*]

[1] Institute of Molecular Biosciences, Mahidol University, Nakhon Pathom, Thailand
[2] Program in Bioinformatics and Computational Biology, Graduate School, Chulalongkorn University, Bangkok, Thailand
[3] Excellence Center for Infectious Diseases, King Chulalongkorn Memorial Hospital, Thai Red Cross Society, Bangkok, Thailand
[4] Division of Infectious Diseases, Department of Medicine, Faculty of Medicine, Chulalongkorn University and King Chulalongkorn Memorial Hospital, Thai Red Cross Society, Bangkok, Thailand
[5] Department of Biochemistry, Faculty of Science, Chulalongkorn University, Bangkok, Thailand

* Correspondence: poochit.non@mahidol.ac.th

This PDF file includes:

Supplementary figures 1 to 4
Supplementary tables 1 to 4

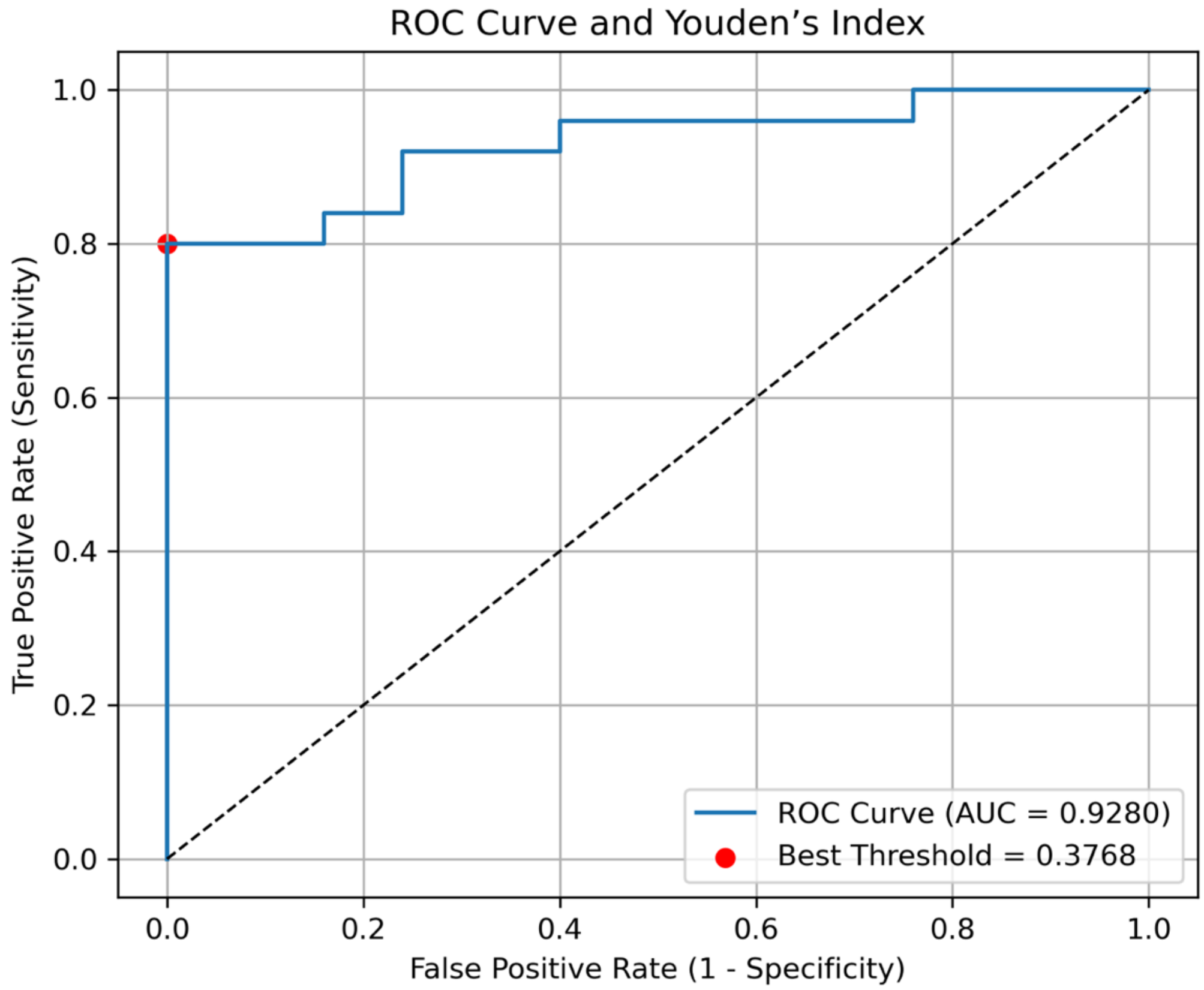

Supplementary figure 1 Receiver Operating Characteristic (ROC) analysis of similarity index threshold for binary classification between *A. baumannii* ATCC 17978 and its CIP-resistant derivatives

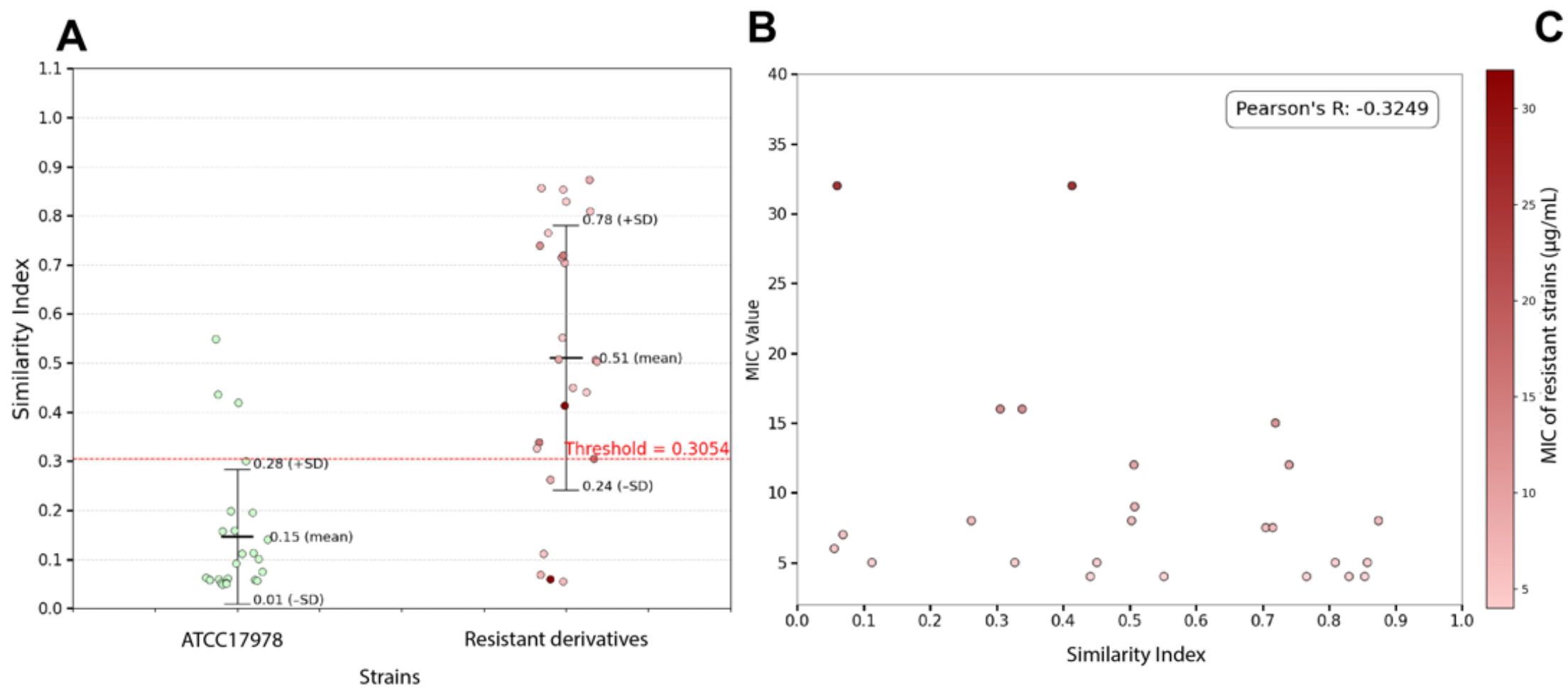

C

Best similarity index threshold	0.3054
Area Under the Curve (AUC)	0.8672
Youden's Index	0.68
Accuracy	0.84
Precision	0.8696
Recall	0.8
F1 score	0.8333

Supplementary figure 2 Performance of the previously reported universal set of 62 features in sensitive and resistant strains classification via degree of morphological changes (A) A summary of similarity index between untreated and CIP-treated of all 25 CIP-resistant strains in comparison with *A. baumannii* ATCC 17978. (B) Scatter plot of MIC values and similarity index analysis of 25 CIP-resistant strains. (C) Precision table of sensitive and resistant classification using similarity index threshold at 0.3054 yielding an AUC of 0.8672, 95% CI [0.7516, 0.9573].

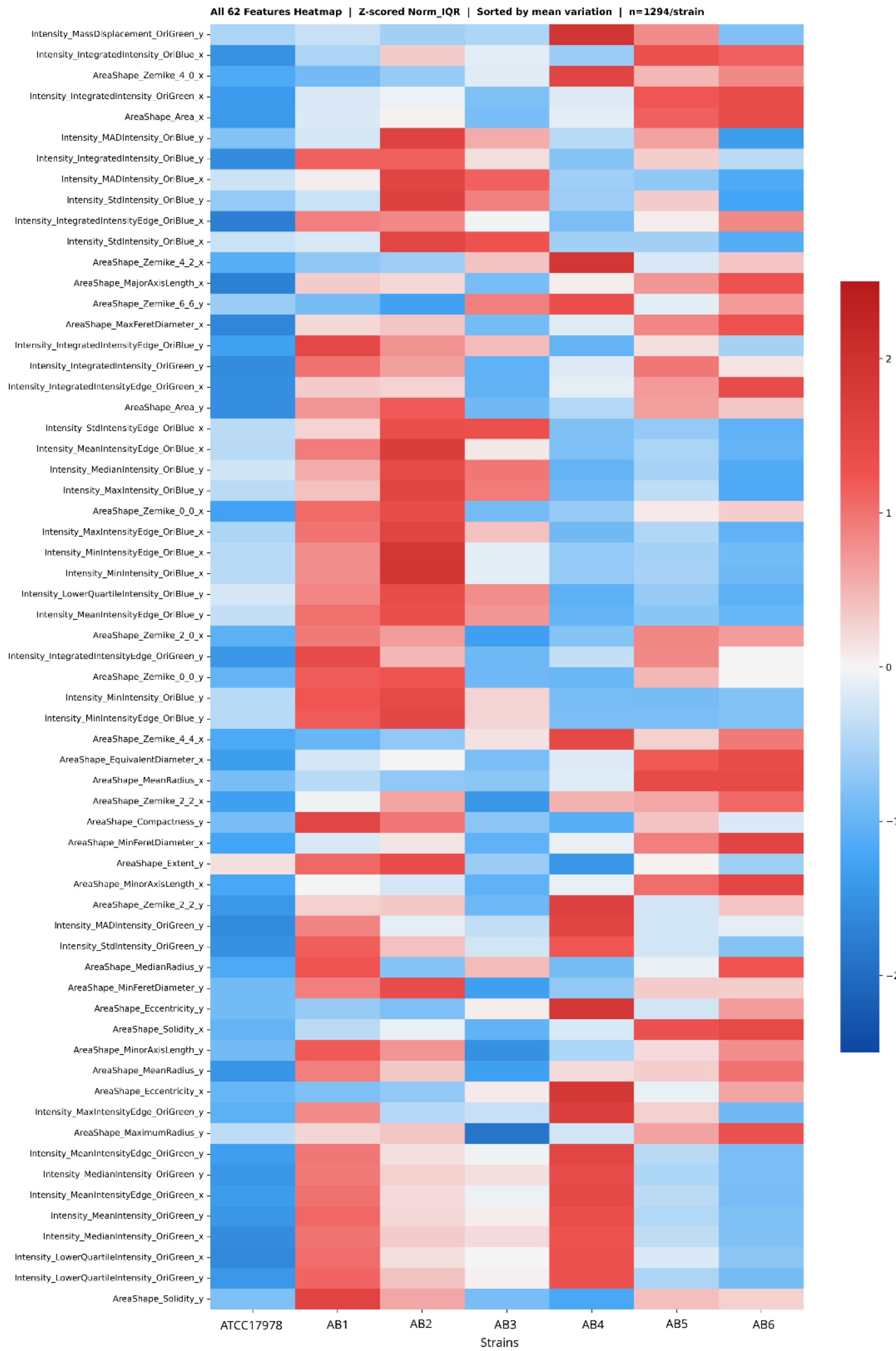

Supplementary figure 3 Heatmap of morphological feature variation profiles

A heatmap displaying the relative heterogeneity of all 62 morphological features of untreated *A. baumannii* strains (n = 1,294 cells per strain). To assess morphological heterogeneity across diverse physical scales, we utilized the Normalized Interquartile Range (Norm IQR), calculated as (Q3 - Q1) /median. For cross-strain comparison, variation scores were Z-scored row-wise; dark red indicates features exhibiting high variability relative to the feature's cross-strain average, while dark blue indicates high population uniformity.

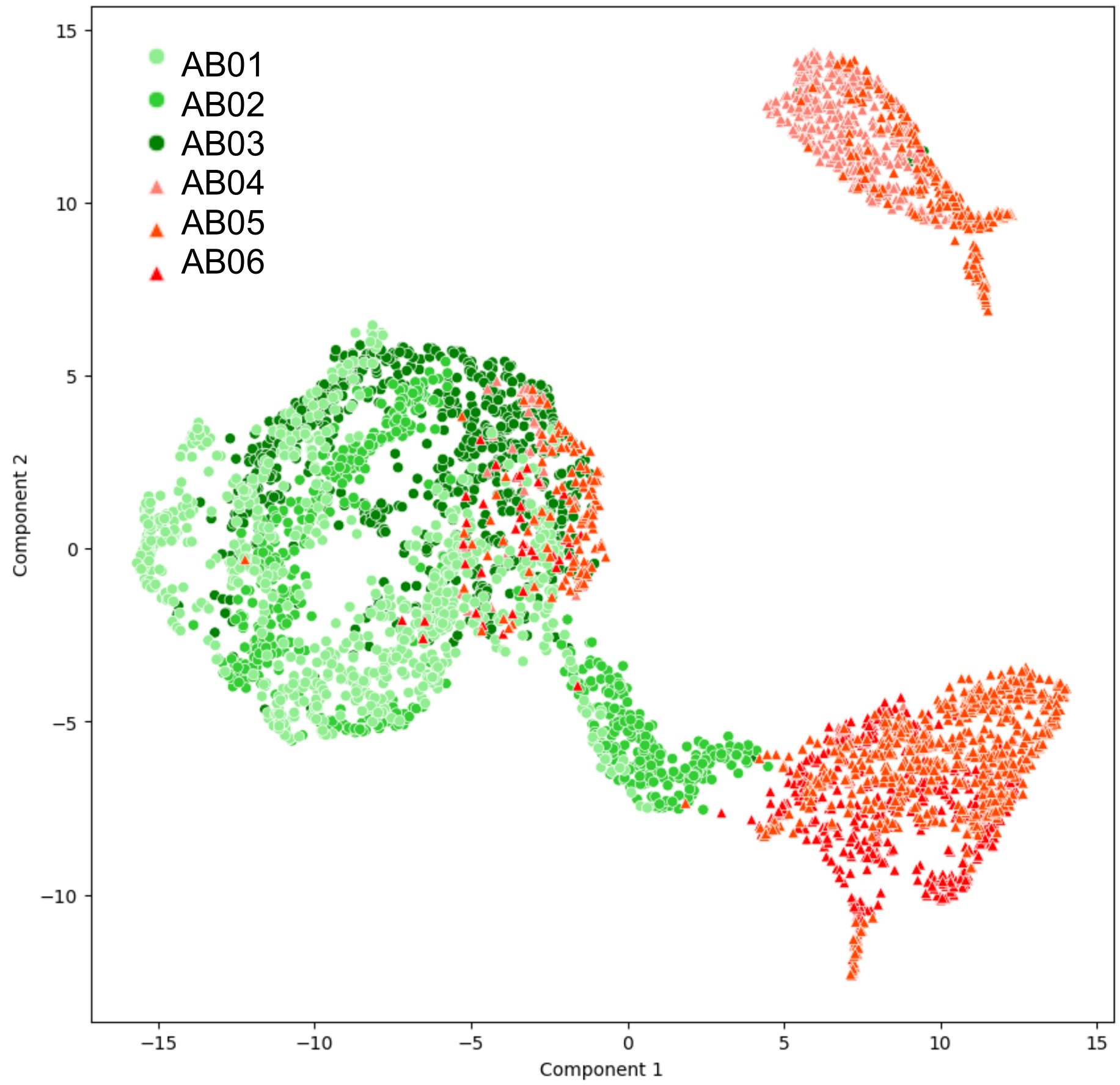

Supplementary figure 4 A PaCMAP plot of untreated populations of AB1–3 (CIP-sensitive) and AB4–6 (CIP-resistant)

Supplementary table 1 Selected 14 morphological features via RFECV-SVM approach with coefficient importance for classifying untreated and CIP-treated cells (x= nucleoid outline, y = membrane outline)

Features	SVM Coefficient Importance
Intensity_MeanIntensityEdge[a]_OriBlue_x	1.686691
Intensity_MedianIntensity[b]_OriBlue_y	1.599402
Intensity_MeanIntensity[c]_OriGreen_x	1.178725
AreaShape_Zernike[d]_2_2_y	0.804233
Intensity_MinIntensity[e]_OriBlue_y	0.755284
Intensity_StdIntensity[f]_OriBlue_y	0.696911
Intensity_MADIntensity[g]_OriBlue_y	0.580975
Intensity_LowerQuartileIntensity[h]_OriBlue_y	0.481282
Intensity_UpperQuartileIntensity[i]_OriBlue_x	0.402215
Intensity_StdIntensityEdge[j]_OriBlue_y	0.32354
Intensity_MaxIntensity[k]_OriBlue_x	0.213617
Intensity_MedianIntensity[b]_OriGreen_x	0.211562
Intensity_LowerQuartileIntensity[h]_OriGreen_x	0.196278
AreaShape_Solidity[l]_x	0.130763

a. MeanIntensityEdge: The average edge pixel intensity of an object.
b. MedianIntensity: The median intensity value within the object.
c. MeanIntensity: The average pixel intensity within an object.
d. Zernike: The Zernike features characterize the distribution of intensity across the object.
e. MinIntensity: The minimal pixel intensity within an object.
f. StdIntensity: The standard deviation of the pixel intensities within an object.
g. MADIntensity: The median absolute deviation (MAD) value of the intensities within the object. The MAD is defined as the median.
h. LowerQuantileIntensity: The intensity value of the pixel for which 25% of the pixels in the object have lower values.
i. UpperQuantileIntensity: The intensity value of the pixel for which 75% of the pixels in the object have lower values.
j. StdIntensityEdge: The standard deviation of the edge pixel intensities of an object.
k. MaxIntensity: The maximal pixel intensity within an object.
l. Solidity: The proportion of the pixels in the convex hull that are also in the object, i.e., ObjectArea/ConvexHullArea.
OriBlue: The fluorescence intensity channel captured from DAPI staining.
Origreen: The fluorescence intensity channel captured from SYTOX Green staining.

Supplementary table 2 CIP-resistant derivatives of *A. baumannii* ATCC 17978 and their MIC against CIP

Strains	MICs (µg/mL)
RS1006	32
RS1007	16
RS1008	32
RS1019	5
RS1028	5
RS1037	16
RS1038	4
RS1044	4
RS1046	12
RS1051	4
RS1054	8
RS1065	8
RS1070	5
RS1072	7.5
RS1078	5
RS1084	6
RS1087	7
RS1091	4
RS1092	4
RS1096	8
RS1102	7.5
RS1105	15
RS1109	12
RS1110	5
RS1115	9

Supplementary table 3 Predicted antibiotic resistance phenotypes of clinical isolates by WGS

Strains	Gene detected	Predicted Resistance Phenotype
AB1	$bla_{\text{OXA-64}}$	Ampicillin
AB2	$bla_{\text{OXA-120}}$	Ampicillin
AB3	$bla_{\text{OXA-259like}}$	Unknown Beta-lactam
AB4	*aac(6')-Ib3*	Amikacin, Tobramycin
	aadA1	Spectinomycin, Streptomycin
	aph(3'')-Ib	Streptomycin
	aph(3'')-Ia	Neomycin, Kanamycin, Lividomycin, Paromomycin, Ribostamycin
	aph(3'')-Id	Streptomycin
	armA	Amikacin, Gentamicin, Tobramycin, Isepamicin, Netilmicin
	$bla_{\text{ADC-25}}$	Unknown Beta-lactam
	$bla_{\text{OXA-23}}$	Imipenem, Meropenem
	$bla_{\text{OXA-66}}$	Imipenem
	catB8	Chloramphenicol
	mph(E)	Erythromycin
	msr(E)	Erythromycin, Azithromycin, Quinupristin, Pristinamycin IA, Virginiamycin S
	sul1	Sulfamethoxazole
	tet(B)	Doxycycline, Tetracycline, Minocycline
	gyrA(S81L) and *parC* (S84L)	Norfloxacin, Ofloxacin, Ciprofloxacin, Levofloxacin
AB5	*aac(6')-Ib3*	Amikacin, Tobramycin
	aadA1	Spectinomycin, Streptomycin
	aph(3'')-Ib	Streptomycin
	aph(3'')-Ia	Neomycin, Kanamycin, Lividomycin, Paromomycin, Ribostamycin
	aph(3'')-Id	Streptomycin
	armA	Amikacin, Gentamicin, Tobramycin, Isepamicin, Netilmicin
	$bla_{\text{ADC-25}}$	Unknown Beta-lactam
	$bla_{\text{OXA-23}}$	Imipenem, Meropenem
	$bla_{\text{OXA-66}}$	Imipenem
	catB8	Chloramphenicol
	mph(E)	Erythromycin
	msr(E)	Erythromycin, Azithromycin, Quinupristin, Pristinamycin IA, Virginiamycin S
	sul1	Sulfamethoxazole
	tet(B)	Doxycycline, Tetracycline, Minocycline
	gyrA(S81L) and *parC* (S84L)	Norfloxacin, Ofloxacin, Ciprofloxacin, Levofloxacin
AB6	*aac(6')-Ib3*	Amikacin, Tobramycin

Strains	Gene detected	Predicted Resistance Phenotype
	aph(3')-Ib	Streptomycin
	aph(3')-Ia	Neomycin, Kanamycin, Lividomycin, Paromomycin, Ribostamycin
	bla$_{ADC-25}$	Unknown Beta-lactam
	bla$_{OXA-23}$	Imipenem, Meropenem
	bla$_{OXA-66}$	Imipenem
	catB8	Chloramphenicol
	tet(B)	Doxycycline, Tetracycline, Minocycline
	gyrA(S81L) and *parC* (S84L)	Norfloxacin, Ofloxacin, Ciprofloxacin, Levofloxacin

Supplementary table 4. Bioinformatics tools, applications, and command lines used in this study

Section A: Nanopore sequencing, quality control, and genome assembly			
**Software**	**Applications**	**Command Lines***	**Software Links**
**Guppy v6.1.7**	Basecalling, demultiplexing, and adapter/barcode trimming	-i [input FAST5 directory]<br>-s [output directory]<br>-c [config file]<br>-x 'cuda:0'<br>--barcode_kits ["SQK-RBK110-96"]<br>--trim_barcodes	Oxford Nanopore Technologies<br>https://nanoporetech.com/document/Guppy-protocol
**pycoQC v2.5.3.0**	Sequencing quality evaluation and visualization	-f [sequencing_summary.txt]<br>-o [output.html]<br>--report_title<br>--min_pass_qual 10<br>--min_barcode_percent 0.1<br>--filter_duplicated	https://github.com/a-slide/pycoQC
**NanoFilt v2.8.0**	Read filtering and trimming for long-read sequencing data	-l 200<br>--headcrop 50<br>--tailcrop 50<br>gunzip -c input.fastq.gz | NanoFilt<br>[options] > output.fastq	https://github.com/wdecoster/nanofilt
**FastQC v0.11.9**	Sequencing quality evaluation and visualization	fastqc input.fastq<br>-o [output directory]<br>--format fastq	https://www.bioinformatics.babraham.ac.uk/projects/fastqc
**NanoPlot v1.40.0**	Sequencing quality evaluation and visualization	--huge<br>--outdir [output directory]<br>--prefix [prefix]<br>--info_in_report<br>--loglength<br>--N50<br>--title [title]<br>--fastq [input.fastq]	https://github.com/wdecoster/NanoPlot
**Flye v2.9.3-b1797**	*De novo* assembly of long-read sequencing data	--nano-hq [input.fastq]<br>--out-dir [output directory]<br>--genome-size 3.4m<br>--iterations 1<br>--scaffold	https://github.com/mikolmogorov/Flye
**Medaka v1.8.0**	Consensus polishing of long-read assemblies using deep learning	medaka_consensus<br>-d [assembly.fasta]<br>-i [input.fastq]<br>-o [output directory]<br>-m r941_min_sup_g507	Oxford Nanopore Technologies<br>https://github.com/nanoporetech/medaka

*Parameters not explicitly specified in the command lines were run using the software default settings.

Supplementary table 4. Bioinformatics tools, applications, and command lines used in this study (continued)

Section B: Genome quality assessment, annotation, and AMR profiling			
**Software**	**Applications**	**Command Lines***	**Software Links**
**Kraken2**	Taxonomic classification of sequencing reads or assemblies	--use-names<br>--db [path to database]<br>--report [filename.report]<br>--unclassified-out [filename.unclassified]<br>--classified-out [filename.classified]<br>--report-minimizer-data [input.fasta] > [output.kraken]	https://github.com/DerrickWood/kraken2
**BUSCO v5.4.3**	Assessment of genome assembly completeness based on single-copy orthologs	-i [input.fasta]<br>-o [output directory name]<br>--mode genome<br>--auto-lineage-prok<br>--out_path [output path]<br>--scaffold_composition	https://github.com/metashot/busco
**CheckM2 v1.0.2**	Assessment of genome completeness and contamination using machine learning	checkm2 predict<br>--input [input directory]<br>--output-directory [output directory]<br>--extension fasta<br>--force<br>--database-path [path to database]	https://github.com/chklovski/CheckM2
**Bakta v1.11.0**	Bacterial genome annotation	bakta<br>--db [path to bakta database v6.0]<br>--output [output directory]<br>--prefix [prefix]<br>--threads 36<br>--keep-contig-headers<br>--verbose [input.fasta]	https://github.com/oschwengers/bakta
**StarAMR v0.5.1**	Antimicrobial resistance (AMR) gene detection	staramr search<br>--plasmidfinder-database-type enterobacteriales<br>-o [output directory]<br>--no-exclude-genes<br>--report-all-blast [input.fasta]	https://github.com/phac-nml/staramr

*Parameters not explicitly specified in the command lines were run using the software default settings.
